# Chronic Ingestion of Sodium and Potassium Bicarbonate, with Potassium, Magnesium and Calcium Citrate Improves Anaerobic Performance in Elite Soccer Players

**DOI:** 10.3390/nu10111610

**Published:** 2018-11-01

**Authors:** Jakub Chycki, Artur Golas, Mateusz Halz, Adam Maszczyk, Michal Toborek, Adam Zajac

**Affiliations:** 1 Department of Sports Training, The Jerzy Kukuczka Academy of Physical Education in Katowice, 40-065 Katowice, Poland; j.chycki@awf.katowice.pl (J.C.); a.golas@awf.katowice.pl (A.G.); mateuszhalz@gmail.com (M.H.); 2 Department of Methodology and Statistics, The Jerzy Kukuczka Academy of Physical Education in Katowice, 40-065 Katowice, Poland; a.maszczyk@awf.katowice.pl; 3 Department of Biochemistry and Molecular Biology, University of Miami, Miller School of Medicine, Miami, FL 33136, USA; mtoborek@med.miami.edu

**Keywords:** supplementation, buffering, speed endurance, team sport athletes

## Abstract

Anaerobic power and anaerobic capacity significantly influence performance in many sport disciplines. These include prolonged sprints in athletics, swimming, or cycling, and other high intensity intermittent sports, such as soccer or basketball. Considering the association of exercise-induced acidosis and fatigue, the ingestion of potential buffering agents such as sodium bicarbonate, has been suggested to attenuate metabolic acidosis and improve anaerobic performance. Since elite soccer players cover from 200 to 350 m while sprinting, performing 40–60 all out sprints during a game, it seems that repeated sprint ability in soccer players is among the key components of success. In our experiment, we evaluated the effectiveness of chronic supplementation with sodium and potassium bicarbonate, fortified with minerals, on speed and speed endurance in elite soccer players. Twenty-six soccer players participated in the study. The subjects were randomly divided into two groups. The experimental group was supplemented with sodium bi-carbonate and potassium di-carbonate fortified with minerals, while the control group received a placebo. The athletes were tested at baseline and after nine days of supplementation. Anaerobic performance was evaluated by the Repeated Anaerobic Sprint Test (RAST) protocol which involved 6 × 30 m max sprints, separated by 10 s of active recovery. Resting, post ingestion and post exercise concentrations of HCO_3_^−^ and blood pH were measured as well as lactate concentration. The current investigation demonstrated a significant increase in RAST performance of elite soccer players supplemented with sodium and potassium bicarbonate along with calcium phosphate, potassium citrate, and magnesium citrate ingested twice a day over a nine-day training period. The improvements in anaerobic performance were caused by increased resting blood pH and bicarbonate levels.

## 1. Introduction

There are numerous sport disciplines in which performance depends to a large extent on anaerobic capacity. These include either single supramaximal efforts, such as prolonged sprints in athletics (200–400 m), swimming (100–200 m), cycling (1000 m) or speed skating (1000–1500 m). On the other hand, many sports are characterized by high intensity intermittent exercise. These include team sports, in which repeated sprint ability is significant for performance or combat sports, where repeated bouts of power are indispensable for success. Both types of exercise may cause disturbances in acid–base balance and fatigue of skeletal muscle. The factors determining fatigue are complex and include both central and peripheral components [1,2]. The decline in performance during exercise that is attributed to the CNS, which integrates input from various body parts, is known as central fatigue. Peripheral components of fatigue include the excessive accumulation of metabolites, of which hydrogen ions (H), potassium (K), and phosphate ions (Pi), as well as the depletion of energy substrates seem to be of greatest significance [3,4]. Single all out efforts lasting approximately 40–60 s cause substantial muscle (6.0–6.4) and blood (6.9–7.0) decreases of pH and lactate concentrations of 22–26 mmol/L and a significant inhibition of glycolytic flux [5]. During team sport games, which may last from 40 min (basketball) to 120 min in an overtime soccer game, decreased repeated sprint ability is primarily attributed to the depletion of muscle glycogen, as the acid–base disturbances are less evidenced, and post exercise lactate concentrations significantly lower [6,7].

Considering the association of exercise-induced acidosis and fatigue, the ingestion of potential buffering agents such as sodium bicarbonate, sodium citrate or potassium bicarbonate has been suggested to attenuate metabolic acidosis and improve anaerobic performance [8,9,10]. Some authors have also suggested chronic using highly alkalized water during periods of intense training and competition to improve hydration and to increase the rate of lactate utilization following anaerobic exercise [11,12].

The ergogenic effects of buffering agents such as sodium bicarbonate have been explored for many decades now. Most empirical data support the benefits of sodium bicarbonate or related substances on exercise performance of different type, duration and intensity [2,13,14,15], however there are reports suggesting no ergogenic effects of buffering supplements as well [16,17,18]. It has been suggested that the discrepancies in results of empirical research with buffering agents are related to: selection of subjects; exercise protocols, especially the intensity, mode and duration of exercise; dosage and timing of supplement ingestion; and the chemical composition of the buffering supplements. As mentioned above, trained and untrained subjects have been included in research [19,20,21,22]; exercise protocols have included single bouts of supramaximal effort [23], intermittent high intensity exercise [24] and skilled based protocols [25,26]. The dosage has usually ranged within 0.3–0.4 g kg^−1^/BM and ingestion time before performance has varied from 60 to 120 min, in single or split doses [20,27,28,29,30,31]. Most studies have used sodium bicarbonate as the only buffering agent in their supplement [28,32,33], while others have tested the combined effects of carbohydrates and sodium bicarbonate [19], creatine and sodium bicarbonate [34], β-alanine and sodium bicarbonate [35,36], and caffeine and sodium bicarbonate [37] on different, sport specific or general exercise modalities. Recently, there are attempts to combine glucose and/or electrolytes with sodium or potassium bicarbonate to increase buffering capacity [7,11]. The majority of authors tested the acute effects of buffering substances on exercise performance [13,38,39], while recently chronic effects of sodium bicarbonate and other buffering agents have also been evaluated with regards to anaerobic performance [15,22]. Considering team sport games, repeated sprint ability test protocols have confirmed positive effects of buffering supplements [11,38], while empirical research with sport specific simulations, including football, soccer, rugby and water polo, have not confirmed ergogenic benefits of sodium bicarbonate [20,40,41].

Soccer is the most popular sport in the world. It is a team sport that involves speed, acceleration, changes of direction as well as numerous technical and tactical activities that require concentration and precision [42]. At the elite level, soccer players perform from 1300 to 1400 different motor activities during a 90 min game. Most specific and general motor activities are executed and repeated at high intensity, causing significant disturbances in acid–base equilibrium and gradual fatigue. A soccer game played at the elite level can elicit up to 85–90% of maximal heart rate, while blood lactate concentrations can reach 7–8 mmol/L at half time and decrease to 5–6 mmol/L after the game, because of glycogen depletion [42]. Depending on the position on the field, players cover from 10,500 to 12,000 m while walking, jogging, running backwards, striding and sprinting [43]. During a game, elite soccer players cover from 200 to 350 m while sprinting, performing 40–60 all out sprints at distances ranging from 5–8 m up to 25–33 m. Considering the above, it seems that repeated sprint abilities in soccer are among the key components determining success. 

This study evaluated the effectiveness of chronic supplementation with sodium and potassium bicarbonate, fortified with potassium, magnesium and calcium citrate and phosphate on speed and repeated sprint ability in elite soccer players.

## 2. Materials and Methods

### 2.1. Subjects

Twenty-six well-trained soccer players, who compete at the elite polish league, participated in the study. The experiment took place during an 11-day camp in Spain, thus training, living and feeding conditions were identical for all participants. The athletes constituted a homogenous group in regards to age, somatic characteristics, as well as aerobic and anaerobic performance (Table 1). The subjects (*n* = 26) were randomly divided into two groups: the experimental group (EG; *n* = 13), which received a complex of independent supplements (sodium bi-carbonate, potassium di-carbonate, calcium phosphate, potassium citrate, magnesium citrate, and calcium citrate (Table 2)), and the control group (CG; *n* = 13), which received a placebo. All subjects had valid medical examinations and showed no contraindications to participate in the study. Subjects were informed verbally and in writing of the experimental protocol, and the possibility to withdraw at any stage of the experiment, and gave their written consent for participation. The study was approved by the Research Ethics Committee at the Academy of Physical Education in Katowice, Poland.

### 2.2. Diet and Supplemental Protocol

Energy as well as macro- and micronutrient intake of all subjects were determined by 24 h nutrition recall 3 weeks before the study was initiated. The participants were placed on an isocaloric (3455 ± 436 kcal/day) mixed diet (55% carbohydrates, 20% protein, 25% fat) prior to and during the investigation. The pre-trial meals were standardized for energy intake (600 kcal) and consisted of carbohydrate (70%), fat (20%) and protein (10%). The participants did not take any medications and substances not prescribed by the supplementation protocol for 3 weeks before and during the study.

The players from the experimental group ingested a single dose of 3000 mg sodium di-carbonate, 3000 mg potassium di-carbonate (6 caps containing 500 mg each), 1000 mg (600 mg + 400 mg) calcium phosphate and calcium citrate, 1000 mg potassium citrate, and 1000 mg magnesium citrate twice a day, 90 min before each practice session. The control group ingested identical capsules containing cornstarch. Supplements were taken with plenty of water (600 mL). The supplementation protocol included an additional dose of di-carbonates and minerals, 90 min before the exercise test protocol and the day before the test. The dose of di-carbonate was chosen according to the literature data, where amounts ranging from 5 to 9 g·day^−1^ are suggested. Such doses have shown significant improvements in buffering capacity with no gastrointestinal distress.

### 2.3. Study Protocol

The experiment lasted 11 days, during which two series of laboratory analyses were performed. The tests were carried out at baseline and after 9 days of supplementation. The study was conducted during the preparatory period of the annual training cycle, when a high volume of work dominated the daily training loads. The participants refrained from exercise for one day before testing to minimize the effect of fatigue.

The subjects underwent medical examinations and somatic measurements. Body composition was evaluated in the morning, between 08:00 and 08:30. The day before, the participants had their last meal at 20:00. They reported to the laboratory after an overnight fast, refraining from exercise for 24 h. The measurements of body mass were performed on a medical scale with a precision of 0.1 kg. Body composition was evaluated using the electrical impedance technique (Inbody 720, Biospace Co., Anaheim, Los Angeles, CA, USA).

Anaerobic performance was evaluated by the Running-Based Anaerobic Sprint Test (RAST) protocol which involved 6 × 30 m maximal sprint efforts, separated by 10 s of active recovery. Infrared photocell gates (Witty, Micro Gate System, Mahopac, New York, NY, USA) were placed precisely 30 m apart. Additionally, two gates were placed at the 5th and 25th m of the sprint distance. The photocell system was used to evaluate the sprint times at 5 and 30 m. The 5 m distance time was considered as starting speed, the 30 m distance evaluated absolute speed, while total time of the 6 × 30 m determined the level of speed endurance and anaerobic capacity. Participants were verbally informed about the time of the rest interval between particular sprints. Before testing, participants were required to complete a 15-min warm-up, which included jogging, dynamic stretching as well as several starts and accelerations. After a 5-min passive rest the participants reported to the starting line and began the RAST protocol on a command. The subjects were instructed to sprint the 30 m distance as fast as they could, decelerate after the finish line and jog back to the starting line for the next repetition. The procedure was repeated until 6 sprints were completed.

### 2.4. Biochemical Assays

To determine lactate concentration (LA), acid–base equilibrium and electrolyte status, the following variables were evaluated: LA (mmol/L), blood pH, pCO_2_ (mmHg), pO_2_ (mmHg), HCO_3_^−^ act (mmol/L), HCO_3_^−^ std, (mmol/L), BE (mmol/L), O_2_SAT (mmol/L), ctCO_2_ (mmol/L), Na^+^ (mmol/L), K^+^ (mmol/L), and Ca^2+^, Mg^2+^. The measurements were performed from fingertip capillary blood samples at rest and after 3 min of recovery. Determination of LA was based on an enzymatic method (Biosen C-line Clinic, EKF-diagnostic GmbH, Barleben, Germany). The remaining variables were assessed using a Blood Gas Analyzer GEM 3500 (Analyzer Premier 3500, GEM, Bedfort, Massachusetts, MA, USA).

### 2.5. Statistical Analysis

The Shapiro–Wilk, Levene and Mauchly’s tests were used to verify the normality, homogeneity and sphericity of the sample’s data variances, respectively. Verifications of the differences between analyzed values before and after di-carbonate and mineral supplementation, between rest and post exercise conditions in the E and C groups were verified using ANOVA with repeated measures. Effect sizes (Cohen’s *d*) were reported where appropriate. According to Cohen’s guidelines, the effect for *r* was established as follows: large effect ≥ 0.5, moderate effect < 0.5 and ≥0.3, and small effect < 0.3 and ≥0.1 [44,45,46,47]. Statistical significance was set at *p* < 0.05. All statistical analyses were performed using Statistica 9.1 (TIBCO Software Inc., Palo Alto, California, CA, USA) and Microsoft Office (Redmont, Washington, DC, USA), and are presented as means with standard deviations.

## 3. Results

The repeated measures ANOVA between the experimental and control group, considering baseline values and the post-intervention period (supplementation) at rest and after exercise, revealed statistically significant results for three variables (Table 1).

Post-hoc tests revealed a statistically significant increase in mean LA post-exercise when comparing the values (from 7.68 to 9.36 mmol/L with *p* = 0.0001) after exercise between the control and experimental group supplemented with di-carbonate and minerals. Similar changes were observed for post-ingestion blood pH (from 7.35 to 7.47 with *p* = 0.0001) and HCO_3_^−^ (from 24.3 to 28.8 mmol/L with *p* = 0.0001) between the control and experimental groups.

**Figure 1:**
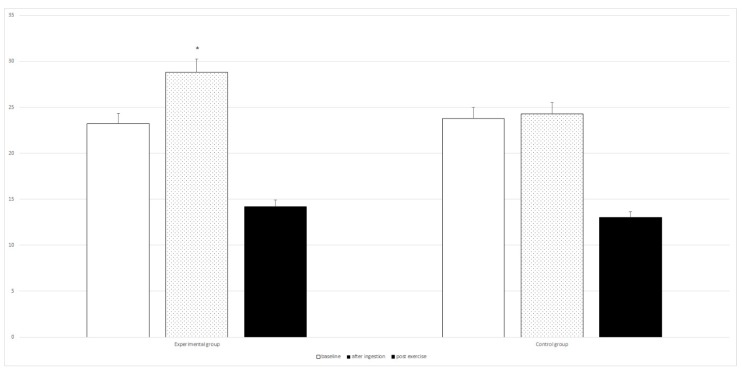
Differences between baseline and post-intervention period in the RAST (6 × 30 m) results for the experimental and control groups. * statistically significant; in experimental group RI = 2.23% (the result improving); in control group RI = 1%.

**Figure 2:**
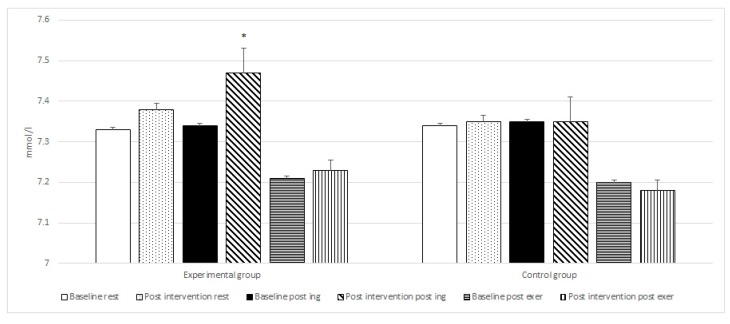
Baseline and post intervention blood pH at rest, post ingestion, and post exercise in the experimental and control groups. * statistically significant.

**Figure 3:**
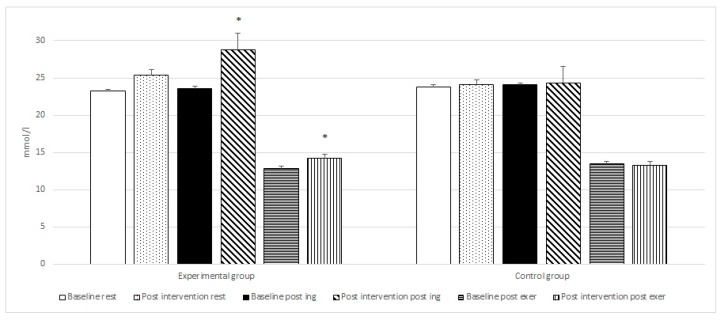
Baseline and post intervention blood HCO_3_^−^ at rest, post ingestion, and post exercise in the experimental and control groups. * statistically significant.

**Figure 4:**
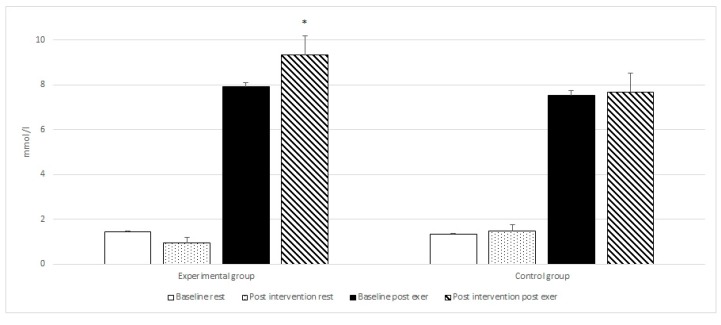
Baseline and post intervention blood HCO_3_^−^ at rest, and post exercise in the experimental and control groups. * statistically significant.

Intragroup analysis with repeated measures ANOVA between the baseline and post-intervention period (di-carbonate and mineral ingestion) at rest, post ingestion and after exercise for the experimental group, revealed statistically significant differences for six variables (Table 2 and Figure 1, Figure 2, Figure 3 and Figure 4). The changes in the control group were not statistically significant.

The post-hoc tests showed a statistically significant improvement in the results of the 6 × 30 m running test (from 25.09 s to 24.53 s with *p* = 0.00001), significant increase in post exercise LA concentration (from 7.94 to 9.36 mmol/L with *p* = 0.00001), as well as an significant increase in post ingestion pH (from 7.38 to 7.47 with *p* = 0.00011), and HCO_3_^−^ values (from 25.42 to 28.81 mmol/L with *p* = 0.00001). Following bicarbonate and mineral supplementation post exercise HCO_3_^−^ concentration increased significantly (from 12.83 to 14.24 mmol/L with *p* = 0.00001), as well as the resting concentration of Mg (from 2.17 to 2.44 mg/dL with *p* = 0.00012).

## 4. Discussion

### 4.1. Ergogenic Effects and Mechanism

The ergogenic effect of sodium bicarbonate and other buffering supplements on exercise performance stems from the reinforced extracellular bicarbonate buffer capacity to regulate acid–base balance during exercise. The oral intake of NaHCO_3_^−^ elevates the concentration of bicarbonate ions (HCO_3_^−^), thus increasing the alkalotic environment in the extracellular fluid compartments [27,28]. The elevated HCO_3_^−^ enlarges the gradient between extracellular and intracellular H^+^, which stimulates the lactate/H^+^ cotransporter [48]. This leads to a greater efflux of H^+^ from intramuscular regions into the extracellular fluid, allowing HCO_3_^−^ and buffering compensatory systems to remove H^+^, thus, increasing pH. Several mechanisms have been proposed to explain how induced alkalosis evokes an ergogenic response to anaerobic exercise, yet there is no consensus among sport scientists. Numerous propositions surrounding both peripherally and centrally driven mediators of fatigue and exercise performance have been investigated [49]. Such mechanisms include the attenuation of exercise-induced arterial oxygen desaturation allowing for enhanced oxygen delivery [50], delayed impairment of muscular contractile properties [51], and augmented glycolytic flux [52]. More recently, research is indicative of an altered neuromuscular response to pre-exercise NaHCO_3_^−^ administration [53,54]. The neuromuscular response that is characterized by a reduced rate of force production declines during isometric contractions after a bout of submaximal exercise [54] and repeated bouts of high intensity exercise [53]. The suggestion therefore is that NaHCO_3_^−^ modifies peripheral indices of fatigue to improve exercise performance. In addition, evidence also has alluded to a central derived contribution to NaHCO_3_^−^ ergogenic effect.

### 4.2. Anaerobic Performance

The current investigation demonstrated a significant increase in anaerobic performance of athletes in the experimental group supplemented with sodium bicarbonate and minerals. The improvements in anaerobic performance following sodium bicarbonate consumption were influenced by significant increases in resting blood pH and bicarbonate concentration.

Anaerobic glycolysis leads to an equal production of lactate and hydrogen ions [55]. Most of the released hydrogen ions are buffered, however, a portion (~0.001%) that stays in the cytosol results in a decrease in muscle pH and impairment of exercise. The rationale for the ergogenic effects of bicarbonate is that the increase in extracellular pH and bicarbonate will enhance the efflux of lactate and H^+^ from the muscle cell [56]. Buffering of protons can attenuate changes in pH and enhance the muscle’s buffering capacity, allowing for a greater amount of lactate to accumulate in the muscle. The results of the current study demonstrated a significant increase in resting blood pH (from 7.38 to 7.47), resting HCO_3_^−^ concentration (from 23.21 to 28.81 mmol/L) and post exercise lactate concentration (from 7.94 to 9.36 mmol/L) in the experimental group supplemented with bicarbonate and minerals.

The concentration of bicarbonate is much lower in the muscle than in the blood (10 vs. 25 mmol/L), and the low permeability of the charged bicarbonate ion precludes any immediate effects on the acid–base status of muscles [57]. These results are in agreement with the view that an appropriate mineral and hydration status is necessary for active bicarbonate ion transport.

Fatigue development during high-intensity intermittent exercise may be caused by a complex interplay between intra and extracellular concentrations, as well as gradients of ions such as K^+^, Na^+^, Cl^−^, H^+^ and Mg^+^ [58,59]. In the present study, no differences were detected in K^+^ and Na^+^ ions, but a significant increase in Mg^2+^ (from 2.17 mg/dL to 2.44 mg/dL). The supplementation with magnesium has been reported to increase muscle strength and power as well as hemoglobin levels [60]. Mg is a cofactor to over 325 enzymatic reactions, and a deficiency of the mineral therefore has many physiological and exercise performance implications. A transient shift of magnesium to the intracellular space during exercise is a probable explanation for a large proportion of the hypomagnesaemia. However, regarding magnesium variations with exercise in red blood cells, dissimilar findings are reported. The magnesium levels in RBC were reported to be increased after several types of exercise [61], and were related to increased metabolic activity during exercise, which would induce a shift of the cation from the plasmatic compartment. Ionized Mg concentration is supposed to be a more sensitive variable than total Mg, giving more reliable information about the status and regulation of major mobilization magnesium pools in the body. However, only limited information about the effects of exercise on the metabolically and regulatory fraction of Mg^2+^ is available [62]. Mooren and co-workers [62] concluded that changes in the fraction of Mg^2+^ should be sufficient to influence intracellular signaling and metabolic processes. Although some explanations have been offered for the compartmental shifts of magnesium, the precise mechanism remains to be clarified. Anaerobic performance enhancement is associated with physiological-regulatory functions of Mg^2+^ within muscle contraction and relaxation. The potential effect is being justified by regulating troponin expression via Ca^2+^ concentration gradients [63], MgATP complex formation optimizing energy metabolism, increasing protein synthetic rate, greater amount of actin-mysoin crossbridges [64] all of which contribute to improved strength and anaerobic metabolism.

Different strategies used for improving buffering capacity of tissues and blood do not allow for a direct comparison. Despite this, there appears to exist an ergogenic effect in response to NaHCO_3_^−^, which may explain the large effect size noted by Tobias et al. [15]. It seems that further work is required to elucidate the mechanism by which sodium bicarbonate and other buffering supplements improve anaerobic exercise performance, although most authors suggest interplay of peripheral and central components [9,14]

The results of our experiment are in line with many other well controlled research projects, which have used repeated high intensity exercise protocols. However, there are some novelties to our study, which should be addressed. First, we used a chronic nine-day supplementation procedure, split into two daily ingestions of a complex containing 3000 mg of sodium di-carbonate, 3000 mg potassium di-carbonate (six caps containing 500 mg each), 1000 mg of calcium phosphate and citrate, 1000 mg potassium citrate, and 1000 mg magnesium citrate. The experimental group of players took the supplement twice a day, 90 min before each practice session. The control group received a placebo that was identical to the buffering supplement. The players were well conditioned before the start of the experiment, and had identical living and training conditions during the study as the experiment was conducted during a preseason camp in Spain. The diet and training loads of the players were controlled and the testing conditions for the RAST were identical for baseline and post intervention measurements. All biochemical evaluations were performed in duplicate in the same laboratory.

## 5. Conclusions

Chronic supplementation with sodium and potassium bicarbonate, fortified with potassium and magnesium citrate, as well as calcium phosphate and calcium citrate, improves repeated sprint ability in elite soccer players. The improvements in anaerobic performance are caused by increased resting and post ingestion blood pH and bicarbonate levels. Although our study is restricted to bicarbonate ingestion combined with chosen minerals, and the statistical power suffers from a low sample size, its results indicate a significant role of magnesium ions in delaying fatigue during high-intensity exercise. The parallel use of minerals and bicarbonate is an innovative aspect of this study and it requires further research. This experiment confirms both acute and chronic buffering effects in elite athletes of sodium and potassium bicarbonate fortified with minerals. Such supplementations protocols can be suggested for competitive athletes before competition or periods of high intensity training to improve anaerobic performance. 

## Figures and Tables

**Table 1:** Statistically significant differences between the experimental and control groups at baseline and after bi-carbonate and mineral supplementation at rest and after exercise.

Variables	*d*	*p*	*F*
LA_post-exercise	0.884	0.0001	802.6
pH_rest	0.780	0.0001	795.5
HCO_3_^−^ rest	0.989	0.0001	1766.9

Note: *d*, effect size; *p*, statistical significance; *F*, value of analysis of variance function; Effect size *r*: *r* ≥ 0.5 is large effect, *r* < 0.5 and ≥0.3 is moderate effect, *r* < 0.3 and ≥0.1 is small effect.

**Table 2:** Statistically significant differences between baseline and post-intervention period at rest, post ingestion, and after exercise for the experimental group.

Variables	*d*	*p*	*F*
6 × 30 m	0.984	0.00001	4812.9
LA post-exercise	0.960	0.00001	4653.1
pH post-ingestion	0.689	0.00011	587.7
HCO_3_^−^ post-ingestion	0.872	0.00001	3541.9
HCO_3_^−^ post exercise	0.798	0.00001	2862.9
Mg^2+^ rest	0.589	0.00012	171.8

Note: *d*, effect size; *p*, statistical significance; *F*, value of analysis of variance function; Effect size *r*: *r* ≥ 0.5 is large effect, *r* < 0.5 and ≥0.3 is moderate effect, *r* < 0.3 and ≥0.1 is small effect.
